# Supplementary material for: Circulating Dopamine Is Regulated by Dietary Glucose and Controls Glucagon-like 1 Peptide Action in White Adipose Tissue
Source: Int J Mol Sci. 2023 Jan 27;24(3):2464. doi: 10.3390/ijms24032464 (PMC9916853; doi:10.3390/ijms24032464)
Supplement: Supplementary file 1 [file ijms-24-02464-s001.zip › ijms-2106657-supplementary.pdf]

Ílium

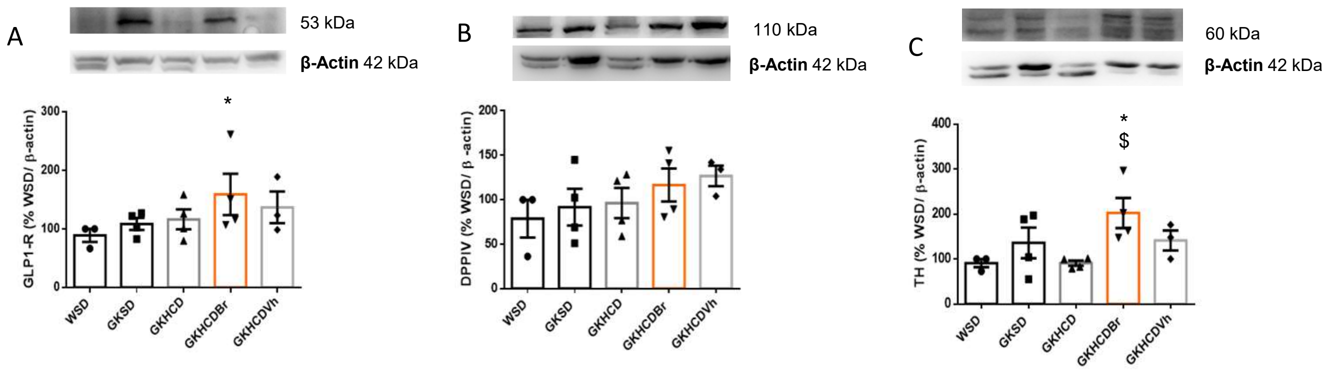

D **GLP1 immunoreactivity**

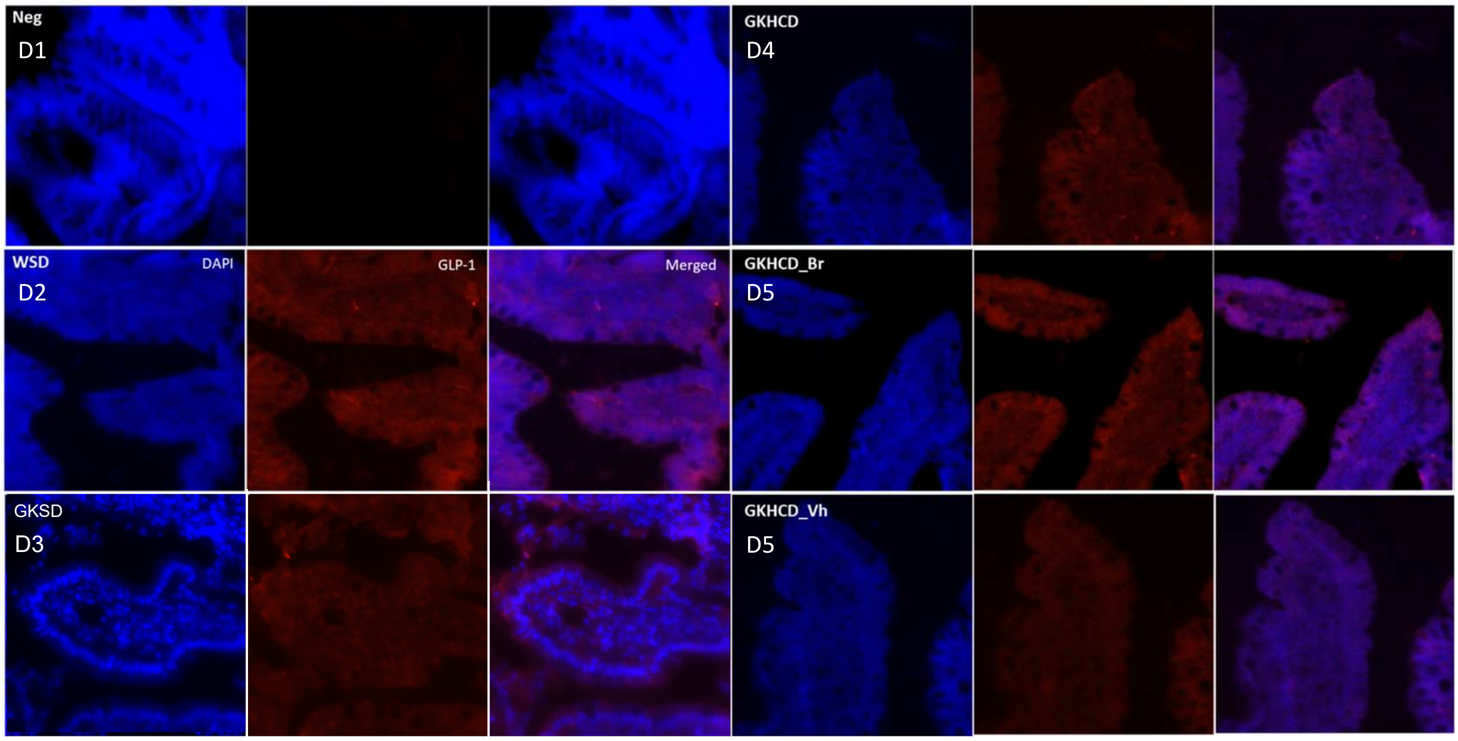

GLP-1 plasma levels

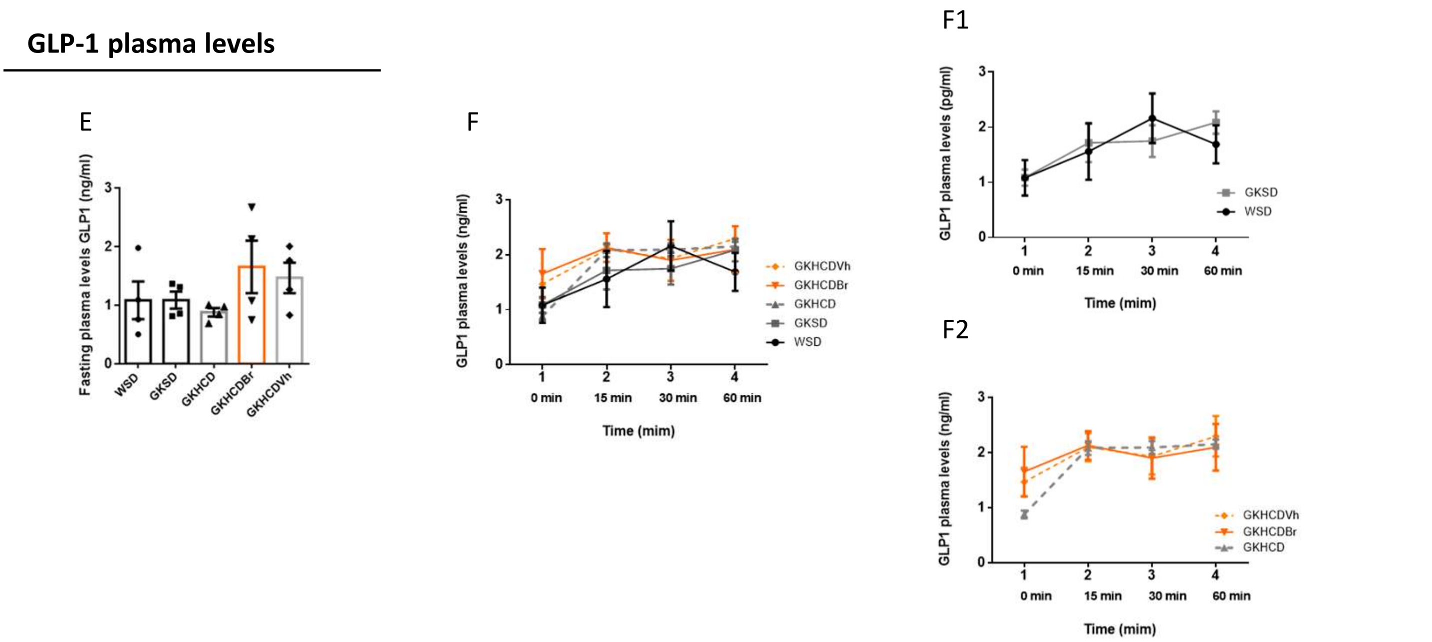

Figure S1. Determination of GLP-1 levels in the intestinal wall of bromocriptine-treated rats. (**A**–**C**) no significant results were obtained for GLP-1， DPP-IV, and TH. (**D**–**F**) no significant changes in postprandial plasma GLP-1 levels were observed. * different from WSD. $ different from GKHCD.
